# Single-Vessel Plume Dispersion Simulation: Method and a Case Study Using CALPUFF in the Yantian Port Area, Shenzhen (China)

**DOI:** 10.3390/ijerph17217831

**Published:** 2020-10-26

**Authors:** Shubin Bai, Yuanqiao Wen, Li He, Yiming Liu, Yan Zhang, Qi Yu, Weichun Ma

**Affiliations:** 1 Department of Environmental Science and Engineering, Fudan University, Shanghai 200433, China; 17210740001@fudan.edu.cn (S.B.); 18110740045@fudan.edu.cn (L.H.); 18210740055@fudan.edu.cn (Y.L.); yan_zhang@fudan.edu.cn (Y.Z.); 2 Intelligent Transportation Systems Research Center, Wuhan University of Technology, Wuhan 430070, China; yqwen@whut.edu.cn

**Keywords:** single vessel diffusion model, SO_2_ emissions, Shenzhen Yantian Port

## Abstract

To study the impact of vessel pollution on the atmospheric environment of the surrounding area, we present a numerical simulation method based on regional emissions inventories. The general spatial resolution is ≥1 km and the temporal resolution is ≥1 h; parameters which are suitable for the study of larger space–time scales. In this paper, the WRF/CALMET/CALPUFF model and Automatic Identification System (AIS) data are employed to develop a single-vessel atmospheric pollution diffusion model. The goal of this research uses existing meteorological models and diffusion models to provide a simulation technology method for studying the diffusion of SO_2_ from a single ship. We take the outgoing phase of ocean-going container vessels in Yantian Port as an example. It can be used to set the position of sensitive receptors near the port area. Simulations are implemented with CALPUFF and the results are compared with data derived from on-site monitoring instrument. The CALPUFF modelling domain covers an area of 925 km^2^ with a grid spacing of 500 m. The simulation results demonstrated agreement with the measured data. The ground concentration contribution value ranged from 10 to 10^2^ μg/m^3^, while the affected area was about 4–26 km^2^ and the high-value area of the ground concentration contribution was distributed within 1–2 km from the ship track. Emissions generated by the vessels represent a considerable contribution to SO_2_ pollution around the harbor areas.

## 1. Introduction

In recent years, maritime transport emissions have been widely considered an important source of air pollution. Vessel emissions could have a negative influence on regional and even global air quality and may provide important contributions to local and regional pollution in areas of high vessel volume along heavily travelled courses [1]. Alexander et al., examined the impacts of PM_2.5_ and NO_x_ emissions originating from vessels in San Pedro Bay, California, and the affected area was found to be within 2–6 km of the port [2]. Two studies have investigated the impact of ship plumes in the moored state on nearby residential areas in busy city ports in Sydney and showed that the relatively high sulfur content (up to 3.5%) in marine fuels led to high SO_2_ emissions [3]. During entry, berthing, and departure, vessel emissions mainly occur in coastal areas and are directly dispersed into the mainland, causing environmental problems affecting both ecosystems and human health [4]. Therefore, research on vessel emissions control has urgent and realistic significance.

One of the world’s major sources of sulfur dioxide (SO_2_) emissions is marine transportation. Sulfur oxide (SO_x_) emissions from shipping activities account for about 60% of SO_x_ emissions globally [5]. As the fuel oil of vessels is high in sulfur, their SO_2_ emissions are relatively high, contributing up to 20% of atmospheric loading in congested areas and their vicinities [1]. The natural environmental impacts of SO_x_ emissions from vessel engines are secondary inorganic aerosol formation and environmental acidification [6]. Moreover, vessel sulfur emissions have been recognized as significant sources of radiative forcing and sulfur cycling over oceans [7]. In 1997, to prevent air pollution from vessels, the International Maritime Organization (IMO) adopted the MARPOL Annex VI regulation [8]. This regulation gradually reduced the limit of sulfur content fuel oil in vessels to less than 0.5% m/m on 1 January 2020 from the 1.0% limit that came into effect on 31 December 2014 [9]. The tightening of sulfur emission restrictions by the IMO has, to some extent, mitigated the environmental implications of SO_2_ emissions from seagoing vessels [10].

At present, the technical methods used to quantitatively study the impact of anthropogenic air pollutants on ambient air quality are air quality models. Many studies have adopted the diffusion model to simulate environmental impacts [6,11,12,13,14,15,16]. Different sorts of Lagrangian particle dispersion models [17], advanced Gaussian dispersion models [12], and puff models [18] have been reported in the literature. The Lagrangian particle model used by Gariazzo in the Taranto area (Italy) has been used to assess the impact of harbor activities on air quality [17]. Merico used a WRF-CAMx modelling system to study vessel emissions and their atmospheric impacts on four port cities: Patras (Greece), Rijeka (Croatia), Brindisi, and Venice (Italy) [13]. Poplawski et al., used the CALPUFF model to investigate the concentrations of sulfur dioxide (SO_2_), nitrogen dioxide (NO_2_), and fine particulate matter (PM_2.5_) from cruise ship emissions in James Bay (Victoria, BC, Canada) and verified that the concentrations simulated by the CALPUFF model were in good agreement with the average concentrations measured by on-site monitoring [19]. Murena carried out a study in Naples (Italy) on the impact of cruise ship emissions on air quality and verified that emissions from vessels using low-sulfur fuel (0.1%) had a low contribution rate to the actual SO_2_ concentration [20].

However, previous numerical simulation studies have been based on the overall emissions of an entire port or were conducted over the time span of a year. Previous studies have simulated the impact of air pollution emissions in an entire port area on regional ambient air quality, but these models cannot be accurately applied to small scales, in terms of time and spatial dimensions. Based on the CALPUFF model, we developed a technical method for single-ship SO_2_ emission diffusion simulation. We use this method to simulate the concentration of sulfur dioxide in the plume discharged from a single ship and compare the time-series of simulated and observed concentrations. Evaluation of the ground-level spatial distribution of the maximum concentration contributed by each simulation grid in the simulation period of atmospheric pollutants emitted by vessels was carried out using CALMET/CALPUFF.

This paper is organized as follows: Section 2 describes the methodology. The first part of Section 3 discusses the simulation test of the meteorological models, while numerical simulations with the single-vessel atmospheric pollution diffusion model are performed in the second part. Section 4 presents the discussion. Finally, conclusions are discussed in Section 5.

## 2. Methods and Data Source

### 2.1. Study Area and Object

**Figure 1:**
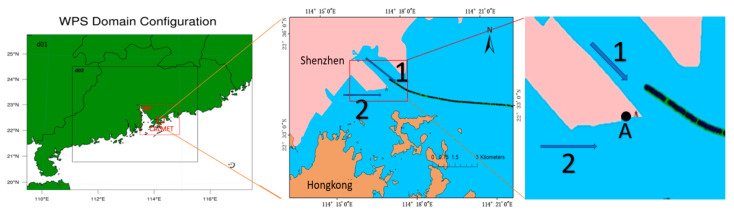
Left: Weather Research and Forecasting Model (WRF) three-layer nesting and CALMET area; Right: Yantian port (Point A is the location of the shore-based monitoring instrument and the numbers 1 and 2 indicate the two waterways when the ship leaves the port).

The study area was a coastal site on the South China Sea in Shenzhen, Guangdong Province in China. Yantian Port is in the south of Shenzhen, adjacent to Hong Kong. A map of it is shown in Figure 1. Shenzhen is located in a sub-tropical—tropical transitional marine climate zone in a low-latitude area, adjacent to a vast ocean. The climate of Shenzhen is, therefore, significantly affected by both the mainland and the ocean. In the past three decades, the average monthly temperature in Shenzhen in summer has been between 25 and 30 °C. In summer (June to August), due to the influence of the ocean air mass and southwestern monsoons, the wind direction is southerly and there is abundant precipitation. June is the peak rainfall period, in which the chance of heavy rain is very high, conducive to the removal of atmospheric pollutants.

Yantian Port is located in Dapeng Bay, southeast of Shenzhen, and backs onto the Pearl River Delta. The seawater in the port is wide and stable, and large ships can dock there. There are special conditions for conducting experiments on the atmospheric diffusion of vessel emissions in Yantian Port. In 2018, Yantian Port had only two channels (Figure 1) and 16 berths. According to the Daily Arrival and Departure Plan for ships provided by the Shenzhen Maritime Safety Administration and observations by field experimenters, the interval between ships entering and leaving the port is basically more than half an hour. At the same time, there is basically only one ship entering or leaving the port; it is rare that several ships enter and leave the port at the same time. Furthermore, not many ships operate at the same time in the port area, such that the monitoring results can be sure to only come from a single vessel.

Emissions from ocean-going vessels have increasingly become a focus in research on the chemical impacts on the climate and oceanic atmospheres [21,22,23,24]. This research is based on ocean-going vessels and does not consider any effects of other kinds of vessels, such as tugs and bulk carriers. Therefore, the outgoing container vessels in Yantian Port were taken as the research object of this study. During loading/offloading operations, auxiliary engines were the main sources of air pollutants in the harbor, where the auxiliary engine was considered equivalent to a fixed-point source. The relatively mature method of simulating the diffusion law of fixed-point source air pollutant emissions, therefore, could be used to calculate its impact on the ambient air quality of the port area. However, in the processes of entering and leaving the port, the ship must be handled as a mobile source. In the arrival stage, the main engine was in a low-load state and the amount of air pollutants emitted was relatively small, while the emissions of the main engine during the departure stage were large. Therefore, we selected the vessel departure stage to study the diffusion law of SO_2_ emissions from a single vessel’s mobile source.

### 2.2. Single-Vessel Diffusion Model

#### 2.2.1. Meteorological Model

We considered that—besides the location and characteristics of the emissions—meteorological conditions are also a key factor in the spatial distribution of the concentration of atmospheric pollutants. The spatial high-resolution meteorological field is closely related to the single-vessel atmospheric diffusion model. In this case, the use of a meteorological model was an important step in studying the diffusion of pollutants from a single vessel.

The meteorological data used as input to the CALPUFF model were 3D fields of meteorological parameters. In this study, meteorological fields were generated by CALMET for a 37 × 25 km Cartesian grid centered on Yantian Port and sub-divided into a 74 × 50 cell grid system with 500 m cell spacing; the CALMET vertical grid system considers 10 levels, from sea level up to 4000 m. The CALMET input data included ground station data and 3D vertically stratified meteorological data (The Weather Research and Forecasting Model, WRF).

In this study, the WRF model version used was 3.6.1, the research center point was set to (22.8° N, 113.48° E), and the simulation area was nested with three layers of grids (see Figure 1). The outermost nested grid had a spatial resolution of 9 km in both longitude and latitude and a grid number of 100 × 79. The second layer covered the majority of Guangdong Province and its coastal areas. The horizontal resolution of the grid was 3 km in both longitude and latitude, and the number of grids was 166 × 145. The inner layer covered the Shenzhen area. The horizontal resolution of the grid was 1 km in both longitude and latitude, the number of grids was 157 × 139, and the number of layers in the vertical direction of the three-layer simulation area was the same (i.e., 27 layers). The main physical parameterization schemes of the WRF model are given in Table 1. Physical parameterization schemes include atmospheric shortwave and atmospheric longwave radiation schemes, atmospheric horizontal and vertical eddy diffusion, land surface schemes, cloud microphysical parameterization schemes, cumulus convection parameterization schemes and boundary layer schemes. Among them, the Dudhia shortwave radiation scheme is derived from the MM5 mode, which simply accumulates the solar radiation flux caused by clean air scattering, water vapor absorption, cloud reflection and absorption. The Noah land surface process parameterization can predict the effects of soil icing and snow accumulation, which improves the ability to deal with urban ground and takes into account the nature of ground emitters. The RRTM long-wave radiation scheme uses a pre-processed comparison table to indicate the long-wave radiation caused by other gases such as water vapor, ozone, carbon dioxide, and the optical thickness of the cloud. The KF (Kain-Fritsch) scheme is a simple cumulus convection model that considers downdraft and humid updraft. The Lin scheme adopted by the cloud microphysical parameterization scheme is a more complex single-parameter scheme, which includes the forecast of clouds, water vapor, rain, snow and cloud ice. In addition, the YSU is the second generation of the MRF boundary layer scheme, which adds a method to deal with the sandwich layer on top of the boundary layer.

#### 2.2.2. Automatic Identification System (AIS)-Based Vessel Emissions Inventories

Emissions inventories provide significant inputs for local, regional, and global atmospheric modelling studies. From a security perspective, the IMO has put a rule in place that international ocean-going vessels must use an Automatic Identification System (AIS) and report certain information (e.g., call number, speed, navigation status, and location) regularly via radio [25,26]. Therefore, it is now possible to build an inventory based on single-vessel activities, which has improved the spatial–temporal resolution of vessel emissions inventories [27]. This article is based on advanced methods of estimating vessel emissions from ship AIS activities [11,28].

In this study, the air pollutant emissions of ships were calculated based on ships and their navigation data (AIS data and basic ship information) and pollutant emission factors. The basic idea is to calculate using the power method of the maximum rated power of a ship’s engines [29]. The pollutant emissions can be expressed using the following formula:

(1) E=P×LF×T×EF,

where *E* represents the emission amount of a certain pollutant (g), *P* represents the maximum rated power of the ship’s engine (kW), *LF* represents the load factor when the ship is sailing, *T* represents the operating time of the ship’s main engine (h), and *EF* stands for pollutant emission factor (g/kWh).

The discharge position of the ship was divided into main engine discharge and auxiliary engine discharge. The engine that provided power for the ship’s navigation was the main engine, while the engine that met the needs of the ship’s power supply and life was the auxiliary engine. Therefore, an estimation method based on the ship’s engine power was adopted [30]. The total emissions of each ship was the sum of the emissions of the main (*Em*) and auxiliary (*Ea*) engines of the ship. Boiler emissions were considered to be negligible and, so, the impact of boilers was not considered in this study:

(2) E=Em+Ea.

The calculation formula for the main engine emissions of a ship is as follows:

(3) Em=P×LF×LLAM×T×EF×CF×FCF,

where *Em* represents the emissions of the main engine, *P* represents the maximum rated power of the ship’s main engine, *LF* stands for the load factor, *LLAM* stands for the main engine low load adjustment factor, *T* represents the operating time of the ship’s main engine, *EF* represents the emission factor corresponding to the main engine, *CF* represents the control factor corresponding to the main engine, and *FCF* stands for the main engine fuel control factor.

The load factor calculation method is as follows:

(4) $$LF={\left(AS/MS\right)}^{3},$$

where *AS* is the current speed of the ship and *MS* is the maximum speed of the ship.

The calculation formula for the emissions of auxiliary engines is as follows:

(5) Ea=P×LF×T×EF×CF,

where *Ea* represents the emissions of auxiliary machinery, *P* represents the maximum rated power of the auxiliary machinery, *LF* stands for the load factor, *T* represents the operating time of the ship’s auxiliary engine, *EF* stands for the emission factor corresponding to the auxiliary engine, and *CF* represents the control factor corresponding to the auxiliary machine.

The various factors involved in the calculation of emissions include main engine and auxiliary engine emission factors, main engine and auxiliary engine control factors, fuel control factors, and main engine low load adjustment factors [30]. All these parameters were derived from [30].

#### 2.2.3. Single Vessel Atmospheric Pollution Diffusion Model Based on CALPUFF

The California Puff (CALPUFF) Modelling System is a multi-species, multi-layer, non-steady-state Lagrangian Gaussian puff dispersion model which contains modules for overwater transport, complex terrain effects, building downwash, coastal interaction effects, simple chemical transformation, and wet and dry removal [6]. This model can simulate the effects of temporally and spatially variable meteorological conditions considering point, line, area, or volume sources [31]. We developed a single-vessel air pollutant diffusion model based on the CALPUFF model.

The CALPUFF model was originally applied to fixed-point sources. In this study, it was modified in order to apply it to moving sources (i.e., vessels). In the harbor, when the model is applied to vessels, the calculations involved are complex due to the fact that the sources are not often stationary and their emissions vary [12]. In this paper, a continuous trajectory of every ship was discretized into a series of time-series geo-referenced spatial points (i.e., a series of points with time characteristics) and it was assumed that, at these specific points, air pollutants were emitted at the time corresponding to the vessel’s navigation (Figure 1). At the same time, as the spatial distance (or time step) of these discrete points was so short that it could be approximately considered that, in such a differential time period, the navigation conditions of the ship and the emission of air pollutants were constant. Based on the above ideas, in the research of this paper, a point source emission file (PTEMARB.DAT) of uneven emissions was created to simulate the real situation of emissions when the ship moved continuously.

The model generates a PTEMARB (i.e., a point source emissions file with arbitrarily varying emissions) file, according to detailed single-ship emissions inventories including the variation of emission rates per several seconds. The discrete point co-ordinates representing the different navigation positions of the ship were used as point source co-ordinates to edit and input the PTEMARB.DAT file during the vessel’s departure. The PTEMARB.DAT file contained the file name, time, UTM co-ordinates, the number of pollution sources in the file, the number of pollutants emitted, the molecular weight of the pollutants, and the temperature of the funnel flue gas outlet (°K), as well as outlet air velocity (m/s), pollutant emission rate (g/s), funnel geometric height (m), funnel diameter (m), and funnel height (m).

All vessels in this experiment generated a point source every ten seconds to form a PTEMARB file. Then, the CALPUFF model was used to simulate the air pollutant diffusion at these discrete points. Finally, the diffusion plumes of these different geographical co-ordinate point sources at different periods were superimposed to obtain the basic law of SO_2_ diffusion in a single vessel.

The study area for CALPUFF single vessel atmospheric diffusion was the same grid as that used in the CALMET domain. Pollutant concentrations were calculated at the 3700 nodes of a 74 × 50 Cartesian receptor grid with 500 m node spacing and at 1 discrete receptor (the air quality monitoring site). The resolution of the simulation time-series was 60 s.

The CALPUFF modelling result was the incremental amount in concentration, rather than the actual ambient concentration, such that the background concentration of pollutants had to be provided for each vessel modelled. The average value which removed the peak segment of shore-based instruments during the simulation period of ship departure was selected as the background concentration. Then, we added the background concentration and the simulated increment together to obtain the simulated environmental concentration, which was compared with the peak concentration observed by the instrument.

#### 2.2.4. Correction of Plume Lifting Height Calculation

During navigation, the lifting height of the plume emitted by the vessel funnel was affected by the relative speed of the vessel and the surrounding air. Consequently, when calculating the lifting height of the plume, the relative wind speed should be considered with the vessel as the reference system. The wind vector simulated by CALMET during the vessel’s departure period was made into a vector difference with the ship’s speed, in order to obtain the relative wind speed:

(6) $$\vec{V_{r}}\;=\;\vec{V_{w}}-\vec{V_{s}},$$

where ${\vec{v}}_{r}$ is the relative wind speed, ${\vec{v}}_{w}$ is the CALMET wind vector, and ${\vec{v}}_{s}$ is the ship’s speed.

The basic plume rise relationships in CALPUFF are based on the Briggs equations [32]. After comparing the simulation results with actual site photos, we determined that the smoke lift height calculated based on the Briggs formula was too large and was quite different from the actual situation. So, we used the national standard Technical Method for Formulation of Local Air Pollutant Emission Standards (GB/T13201-91), issued jointly by the State Environmental Protection Administration and the State Technical Supervision Bureau of China, to give the calculation formula for the flue gas lifting height. When the flue gas heat release rate satisfied *QH* ≥ 2100 kJ/s and the difference between the flue gas temperature and the ambient temperature satisfied Δ*T* ≥ 35 K:

(7) $$\Delta h=n_{0}\times Q_{H}^{n_{1}}\times h_{s}^{n_{2}}\times {\bar{u}}^{-1},$$

where Δ*T* represents the difference between the flue gas temperature and the air temperature, *n*_0_ represents the surface condition coefficient, *n*_1_ represents the flue gas heat release rate index, and *n*_2_ represents the chimney height index. All the parameters, coefficients, and adjustment factors involved were derived from the national standard Technical Method for Formulation of Local Air Pollutant Emission Standards (GB/T13201-91).

#### 2.2.5. The Statistical Evaluation for the WRF and CALMET

Wind direction and wind speed have been found to be the two most important meteorological factors affecting the diffusion of pollutants in the CALPUFF model. In order to evaluate the simulation effect of the WRF on the wind field, two indicators—wind direction and wind speed—were selected. The measured data from Beizaijiao (BZJ), Yantiangang (YTG), and Shatoujiao (STJ) were compared with the WRF results, in order to evaluate the performance of the model. The Root Mean Square Error (RMSE), Mean Bias (MB), Correlation Coefficient (COR), and Index of Agreement (IOA) were selected for the wind speed test. The wind direction is the prevailing wind direction, not the absolute wind direction and, so, it cannot be tested by the root mean square error, mean bias, correlation coefficient, and index of agreement. The statistical calculation of the wind direction was determined by finding the actual angle between the simulated and observed wind directions. If (X_p_ − X_o_) > 180°, then (X_p_ − X_o_) = |(X_p_ − X_o_) − 360°|. The expressions of the selected parameters are as follows:

(8) $$\mathrm{MB}=\frac{1}{N}\sum_{i=1}^{N}(X_{p}-X_{o}),$$

(9) $$\mathrm{RMSE}=\sqrt{\frac{1}{N}\overset{N}{\underset{i=1}{\sum }}(X_{p}-X_{o}{)}^{2}},$$

(10) $$\mathrm{IOA}=1-\frac{\sum_{i=1}^{N}(X_{p}-X_{o}{)}^{2}}{\sum_{i=1}^{N}{(\left|X_{p}-\bar{X_{o}}\right|+\left|X_{o}-\bar{X_{o}}\right|)}^{2}},$$

(11) $$\mathrm{COR}=\frac{\sum_{i=1}^{N}(X_{p}-\bar{X_{p}})(X_{o}-\bar{X_{o}})}{\sqrt{\sum_{i=1}^{N}(X_{p}-\bar{X_{p}}{)}^{2}(X_{o}-\bar{X_{o}}{)}^{2}}},$$

where $X_{p}$ represents the simulation value, N is the sample size, $X_{o}$ represents the observation value, and $\bar{X_{o}}$ and $\bar{X_{p}}$ represent the average values of the observation and simulation results, respectively.

### 2.3. Data Source

The Yantian vessel plume experiment was conducted from 22 June to 5 July 2018, about 2 km off the coast of Shenzhen. At point A of Figure 1, a shore-based monitoring instrument was set up. In this way, all vessels passed through point A when leaving the port. The experimental monitoring system included a SO_x_ gas analyzer. The SO_x_ gas analyzer used pulsed fluorescence technology to measure sulfur dioxide up to 100 ppm, with accuracy of 1 ppb.

The system used remote control and transmission means to complete the power-on and power-off control of related equipment in the shore-based environmental quality monitor, complete data collection, and store and remotely transmit environmental quality monitoring data. The shore-based monitoring instrument was mainly composed of cabinets, UPS power supplies, industrial control hosts (including data acquisition and storage equipment, power control modules, and remote control and data transmission modules), temperature adjustment equipment, fans, desalination equipment, chimneys, and other supporting structural equipment.

The monitoring instrument measured data including SO_2_, humidity, temperature, barometric pressure, precipitation, wind speed, and direction. In this study, the above-mentioned monitoring data was mainly used for comparison with model simulation data, in order to assess the impact of vessel air pollutant emissions on ambient air quality.

**Figure 2:**
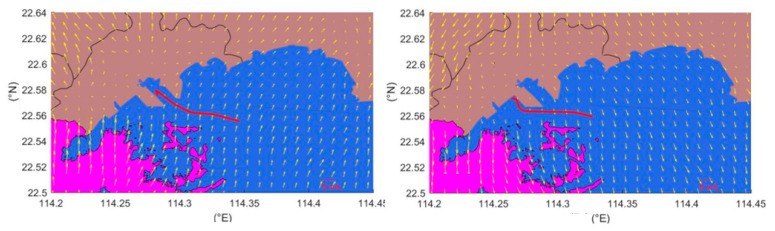
Wind field and ship departure track (the red line represents the ship’s departure trajectory and the vector arrows represent the wind direction and speed).

Based on the data obtained during the Yantian Port experiment from 22 June to 5 July 2018, the SO_2_ concentration peak periods of shore-based instruments were compared with the daily entry and exit plan and the AIS data records. This method was used to find outgoing ships during the response time of the instrument. CALMET simulated the wind direction results combined with the trajectory of the ship. However, based on the CALMET simulated wind direction results and repeated comparisons with ship trajectories, it was determined that the plumes of some ships selected by the aforementioned method during the departure period would not have spread to the location of the shore-based instrument. According to the location of the shore-based instruments, ships whose smoke plume could not spread to shore-based instruments were divided into two categories. As shown in Figure 2, when the wind direction was northerly, if the ship left the port from the left channel, the smoke plume was not able to spread to the shore-based monitoring point; similarly, when the wind direction was southerly, if the ship left the port from the right channel, the smoke plume was not able to spread to the monitoring point, either. It can be seen, from Figure 2, that using the wind direction simulated by CALMET and the trajectories of these ships, the shore-based monitoring point selected in this experiment was located in the upwind direction of the ship’s emissions and would not be affected by the emissions of these ships. The test thus failed to simulate the concentration contributions of these ships at the shore-based monitoring point. Therefore, the above-mentioned ships were not considered in the follow-up study, but only other ships that contributed to the concentration of the shore-based monitoring point in the CALPUFF test were considered. Part of the related information is listed in Table 2.

The characteristics of vessel emissions (i.e., stack height, plume temperature, stack diameter, and exit velocity) might be responsible for pollutant distribution in the port. Therefore, we designed a survey to investigate the basic parameters of each ship. Detailed information on the capacity of engines and the funnel parameters of all ships obtained from the survey were applied in the calculations for each ship. AIS data were provided by the Shenzhen Yantian Maritime Safety Administration.

Land-use data were selected from the Global Land Cover Characteristics (GLCC) database for Eurasia–Asia provided by the U.S. Geological Survey (USGS) at a 1 km resolution. Terrain elevation data were downloaded from http://dds.cr.usgs.gov/in USGS90 format at a resolution of 90 m. Data from ground weather stations were obtained from three sites: Yantian Port, Shatoujiao, and Beizijiao. These data came from the Shenzhen Meteorological Data Network (http://data.121.com.cn/wdn// view/qhdata/).

## 3. Case Studies and Results

### 3.1. The Statistical Evaluation for the WRF and CALMET Models

#### 3.1.1. The Statistical Evaluation for the WRF

Table 3 summarizes the simulation results for the test statistics of the wind speed during the simulation period. The average deviation of the simulated wind speed (MB) was between 2.01 and 2.58 m/s, the RMSE was between 2.18 and 2.82 m/s, and the average deviation of the simulated wind speed (MB) was 2.27 m/s. The simulated wind speed at Yantian Port Station had the smallest MB, at 2.01 m/s. In the simulation, the wind speed basically reflected the daily variation characteristics of the wind speed in the port area, but there was some overestimation.

#### 3.1.2. The Statistical Evaluation for the CALMET

The CALMET results used hourly data from two local weather stations (Shatoujiao and Beizaijiao) to assimilate the wind field from the WRF modelling. In order to better evaluate the simulation effect of CALMET, the wind speed measured by another weather Station (Yantiangang) were selected for comparison with the CALMET results. Table 4 summarizes the test statistics. CALMET was used to further revise the simulation results of WRF. The average error (MB) of CALMET for the wind speed simulation was 1.83 m/s, the root mean square error (RMSE) was 2.02 m/s, and the correlation coefficient was 0.46. These results indicate that the wind speed was closer to the measured data overall, and that the simulation effect was greatly improved. However, due to the large fluctuations in the daily change of wind speed and the influence of local topography, CALMET sometimes overestimated the simulated wind speed. Overall, the simulation results were greatly improved.

Comparison and analysis between the measured data and the simulation results showed that the model had a good effect on the meteorological field during the simulation. CALMET was able to better reproduce the wind field conditions during the simulation period and provided a reliable meteorological background field for the subsequent pollutant diffusion simulation.

### 3.2. Simulation of Single Ship Exhaust Pollutant Diffusion

Using the single-vessel atmospheric diffusion simulation method proposed in Section 2.2.3 and the flue gas lift height correction method proposed in Section 2.2.4 of this article, the spread of SO_2_ emissions from ships during the test period was numerically simulated. During the ship’s departure stage, we also calculated the temporal and spatial distribution of the contribution of SO_2_ emissions to the experimental receptors and the pollutant concentration contribution in the port area.

#### 3.2.1. Comparison of Simulation Results with Shore-Based Monitoring Data

A comparison between the simulation and observation results was carried out for typical vessels during the experiment, in which the concentration obtained by numerical simulation was compared with the shore-based observation concentration at the corresponding position. Analysis of the observation and simulation data for typical ships at departure showed that the simulation results agreed well with the observation data. Due to space limitations, only one ship’s simulation results were selected for analysis in the text. Detailed analysis of one ship—VESSEL B—was conducted, as detailed in the following. See Appendix A for the results of several other vessels.

**Figure 3:**
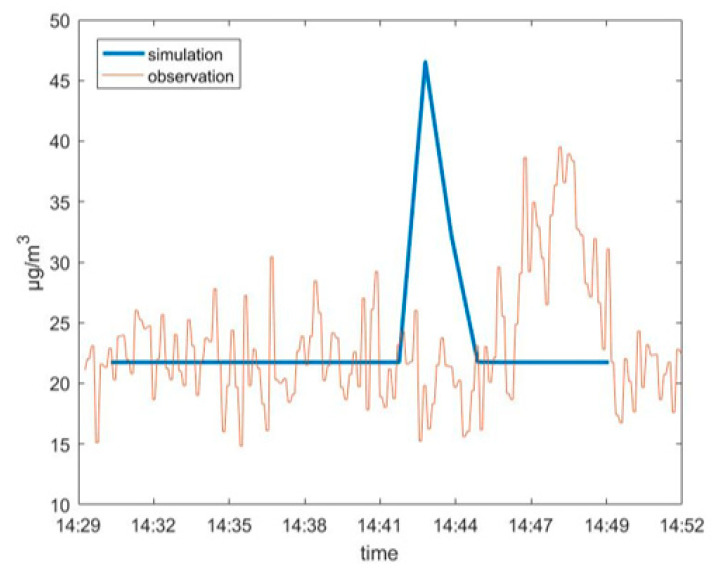
Vessel B’s SO_2_ time-series comparison of simulations and observations.

Figure 3 shows Vessel B’s SO_2_ time-series comparison of simulations and observations. The blue line represents the simulation curve, and the brown line represents the observation curve. The simulation period for VESSEL B was 14:30–14:50 on 24 June 2018. During the simulation period, the observed value has been fluctuating up and down. The CALPUFF modelling result was the incremental amount in concentration, rather than the actual ambient concentration, such that the background concentration of pollutants had to be provided for each vessel modelled. The average value which removed the peak segment of shore-based instruments during the simulation period of ship departure was selected as the background concentration. Then, we added the background concentration and the simulated increment together to obtain the simulated total concentration, which was compared with the peak concentration observed by the instrument. The simulated peak value (the simulated concentration refers to the sum of the concentration contribution (increment) calculated by the single-ship atmospheric diffusion model and the background concentration, the same below) was 47.2 μg/m^3^ and the monitored peak value was 38.7 μg/m^3^. The observed peak of VESSEL B occurred at 14:48, while the simulated peak occurred at 14:43 (i.e., 5 min earlier than the monitored peak).

For all ships in this case study (Table 2), the simulated concentrations were in the range of 20–60 μg/m^3^, while the observed concentrations were in the range of 10–70 μg/m^3^ (see Appendix A). During departure, the observed concentration showed a notable response to the vessel passing through the land-based observation point. For these typical ships, the simulated peak value was generally similar to the monitored peak, in terms of value. The simulated peak occurrence of seven ships was ahead of the monitored peaks by about 3–7 min. There were three ships whose simulated peak time lagged behind the monitored peak by 5–15 min. There was one ship whose simulated peak occurred at basically the same time as the monitored peak.

Some plumes passed through the shore-based instrument and the instrument captured the peak response. By comparing the simulation results and observation data, it was found that the simulated concentration peaks and monitored concentration peaks of some vessels did not appear at the same time. This may have been related to a certain deviation between the simulated wind speed and the actual wind speed.

Table 5 lists the average actual wind speed measured by the monitoring instrument during the simulation period and the hourly simulated wind speed in the CALPUFF grid at the same position (i.e., of the monitoring instrument).

From Table 5, it can be seen that, when the simulated wind speed was greater than the observed wind speed (i.e., the actual wind speed), it was possible that the simulated concentration peak appeared earlier than the observation. When the simulated wind speed was less than the observed wind speed (i.e., the actual wind speed), it was possible that the simulated peak lagged behind the observation.

#### 3.2.2. Impact on the Atmospheric Environment

Throughout our experiment, the simulation results matched well with the observed data. We conducted a detailed analysis of one ship, Vessel B. See the Appendix A for the statuses of other vessels.

**Figure 4:**
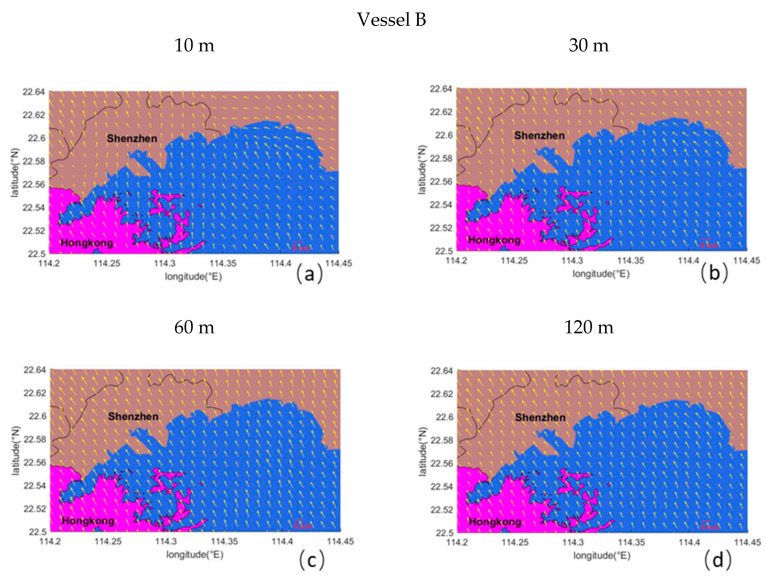

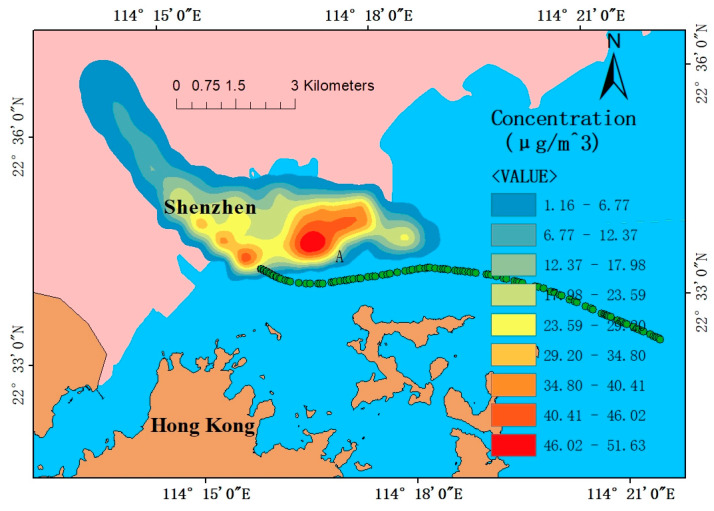
Wind field at different heights at the departure stage of Vessel B and the spatial distribution of the maximum concentration of each simulation grid during the simulation period. (**a**) 10 m, (**b**) 30 m, (**c**) 60 m and (**d**) 120 m.

Figure 4 shows the wind field at different heights at the departure stage of Hyundai Hongkong and the spatial distribution of the maximum concentration in each simulation grid. The wind direction dominated the direction of plume diffusion, where the ground concentration distribution correspondingly extended downwind. The magnitude of the wind speed had an effect on the spatial distribution of the ground concentration, affecting the dilution of the plume.

As shown in Figure 4, when Vessel B left the port, the wind direction was southeast, such that the ground concentration distribution was located to the northwest of the ship track. During the voyage from west to east, the emitted plume gradually lifted to the effective source height, while the ground concentration diffused further in the northwest direction. The range of influence of the maximum concentration contribution was about 20 km^2^, mainly in the Yantian District. The high-value zone appeared within about 1 km of the ship track, where the extreme value concentration contribution was 51.63 μg/m^3^. The high-value range was small (less than 2 km^2^). At the start of the departure phase, the impact of the vessel on the area near the terminal cannot be ignored.

## 4. Discussion

There were certain errors in the model in this experiment. According to the experience of meteorological field simulation in this article, the inconsistency of the peak time was mainly due to a certain amount of deviation between the wind speed and direction simulated by WRF/CALMET and the actual situation. If the simulated wind speed was smaller than the observed wind speed, the simulated peak time could lag behind the observation; meanwhile, if the simulated wind speed was larger than the observed wind speed, the simulated peak time could be ahead of the observation. Furthermore, when the monitoring instrument was located in the downwind direction of the simulation, it was more conducive to the rapid spread of the plume into the range that the instrument could recognize. In order to further reduce the meteorological field error, a more accurate meteorological model should be developed or selected, such as a computational fluid dynamics (CFD) model. The underlying surface of the port area was complex, which was the interface between water and land. Therefore, the use of CFD models to carry out detailed meteorological field simulation could be a research approach. The ships selected for this experiment and numerical simulation study were ocean-going container ships. Other different types of ships (such as tugboats) emissions, and emissions from the operation of vehicles and other operating machinery in the port area had not been considered. Hence, the phenomenon of small peaks or individual multi-peaks in a few experimental periods of shore-based monitoring instruments could not be well explained.

Due to the position of the shore-based instruments, only southerly and eastly winds could disperse plumes to the position of the instruments. If equipment conditions (e.g., power supply) allow, more observation instruments should be added in different directions around the channel. Multiple instruments could be used for monitoring under different meteorological conditions, in order to obtain more effective real-time observation data. In this way, a large number of data samples could be gathered to satisfy further statistical requirements, which would be conducive to further testing the model. We hope to establish large-scale, long-term observations, such as one quarter, one year, or multiple years of continuous observation. In this way, we could correct the model results by obtaining seasonal or interannual vessel air pollution effects on the air quality of the port. In the diffusion model, further consideration need to be given to the impact of mechanical turbulence near the discharge port caused by the complexity of the ship’s superstructure, and the forward and backward tilt (not upward) of the funnel exit on the plume diffusion.

## 5. Conclusions

In this paper, we took Yantian Port in Shenzhen as an example, established a single-ship atmospheric pollutant diffusion model based on the CALPUFF model, and simulated the impact of the single-vessel departure phase on air quality. We also used a combination of the WRF/CALMET atmospheric models to simulate the meteorological field of Yantian Port in Shenzhen. First, the WRF model was used to simulate the meteorological field at the regional scale. Then, the CALMET diagnostic model was used and ground station meteorological observation data from Yantian Port, Beizijiao, and Shatoujiao were introduced to modify the WRF result. PTEMARB.DAT point source files with dynamic emission parameters were generated by using an emissions inventory based on AIS data. CALPUFF was used to simulate the migration and diffusion of the plume emitted by a single vessel into the atmospheric environment, along with the spatial and temporal distribution of its contribution to the surrounding areas. Finally, the single-vessel diffusion model was used to simulate the impact of ocean-going vessels at the departure stage on the ambient air quality. On a small scale (i.e., in the port area), the study of single-vessel mobile sources provides a new approach and technical method for calculating the impact of ship emissions on ambient air quality.

We analyzed the environmental impact of a single vessel’s SO_2_ by combining wind fields at different heights (i.e., 10, 30, 60, and 120 m). The plume diffused along the trajectory of the ship to the periphery and the main diffusion direction was downwind. The ground concentration contribution value ranged from 10 to 10^2^ μg/m^3^, the affected area was about 4–26 km^2^, and the high-value area was distributed within a range of 1–2 km from the ship track. Vessel emissions from the southeast wind had a greater impact on the Shenzhen land area, where most of the concentration contribution from ship emissions under a southwest wind occurred at sea. Our model results show that ships contribute considerably to SO_2_ pollution around harbor areas. During the departure phase, ocean-going container vessels may have an important adverse impact on the short-term air quality of areas near the port.

## Figures and Tables

**Table 1:** Main physical parameterization schemes of the model.

Physical Parameterization Scheme Setting	
Short wave radiation	Dudhia
Land surface process	Noah
Long wave radiation	RRTM
Cumulus convection	Kain–Fritsch
Microphysics	Lin
Boundary layer	Yonsei University (YSU)

**Table 2:** Vessel funnel parameters.

Number	Vessel *	Quantity of Funnels	Funnel Height (m)	Average Exhaust Temperature (°C)	Quantity of Main Engine Funnels	Diameter of the Main Engine Funnel (m)	Quantity of Auxiliary Engine Funnels	Diameter of the Auxiliary Engine Funnel (m)
1	G	7	44.05	60	1	3	4	1.1
2	E	7	44.04	400	1	2.866	5	0.71
3	F	7	38.8	320	1	2.1	4	0.6
4	A	6	46	350	2	2.44	2	1
5	B	2	34.69	370	1	3.2	1	3.2
6	C	8	39.04	200	1	1.38	4	0.98
7	D	7	31.7	225	1	1.712	4	0.508/0.68
8	K	6	33.7	157	1	2.2	4	0.5
9	H	1	33.68	110	1	2.6	-	-
10	I	3	38.8	250	1	2.3	1	0.6
11	J	1	39	47	1	2.2	-	-

* We have hidden the real ship names and replaced them with letters, and the symbol “-” means missing data.

**Table 3:** Test statistics of the WRF results.

Wind Speed	MB	COR	RMSE	IOA
Beizaijiao (BZJ) wind speed	2.18	0.58	2.35	0.83
Yantiangang (YTG) wind speed	2.01	0.56	2.18	0.86
Shatoujiao (STJ) wind speed	2.58	0.69	2.82	0.87

**Table 4:** Test statistics of the CALMET results.

	MB	COR	RMSE	IOA
Wind speed	1.83	0.46	2.02	0.91

**Table 5:** Comparison of wind speeds: CALMET and actual measurement.

Serial Number	Classification	Time	Vessel	Simulation (m/s)	Observation (m/s)
1	Simulation peaks occurrence was ahead of observation peaks	24 June 2018 0:30–0:50	A	4.39	2.04
2		24 June 2018 14:30–14:50	B	4.10	3.89
3		25 June 2018 0:25–0:55	C	5.10	4.13
4		5 July 2018 1:30–2:00	D	2.09	1.19
5		25 June 2018 8:05–8:35	E	5.67	3.62
6		25 June 2018 21:20–21:40	F	1.15	1.30
7		25 June 2018 0:25–0:45	G	5.10	3.89
8	Simulation peak occurrence was the same as the observation peak	30 June 2018 14:30–14:59	H	2.26	2.89
9	Simulation peaks occurrence lagged behind observation peaks	29 June 2018 10:00–10:30	I	3.5	4.34
10		1 July 2018 8:50–9:20	J	2.10	3.18
11		26 June 2018 4:30–4:59	K	1.15	2.39

**Figure A1:**
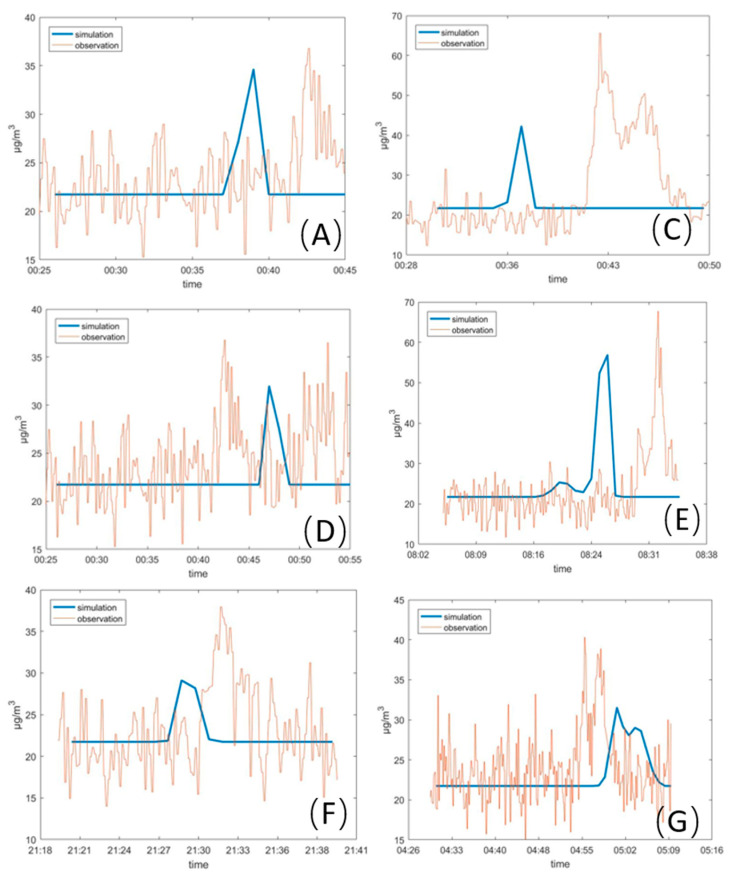

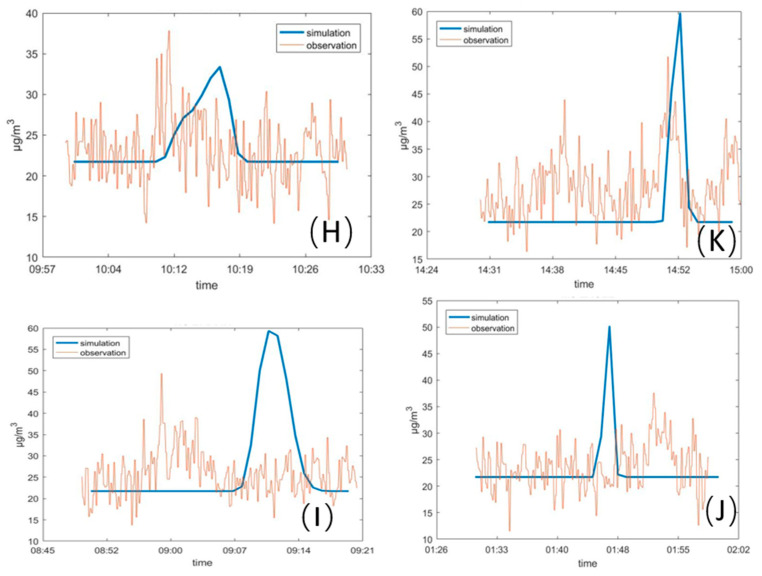
Time-series comparison of simulations and observations of typical vessels. (**A**) vessels A, (**C**) vessels C, (**D**) vesselsD, (**E**) vessels E. (**F**) vessels F, (**G**) vessels G, (**H**) vessels H, (**K**) vessels K, (**I**) vessels I, (**J**) vessels J.

**Figure A2:**
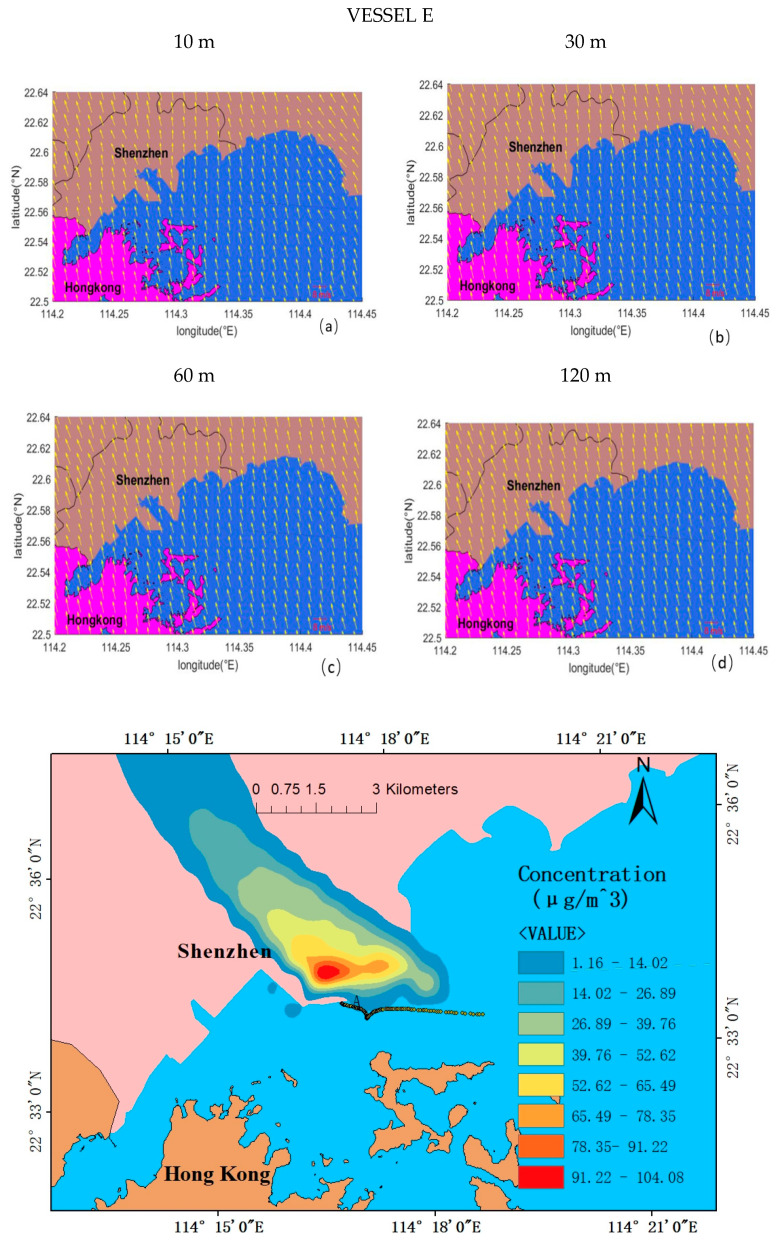
Wind field at different heights at the departure stage of Vessel E and the spatial distribution of the maximum concentration of each simulation grid during the simulation period. (**a**) 10 m, (**b**) 30 m, (**c**) 60 m and (**d**) 120 m.

**Figure A3:**
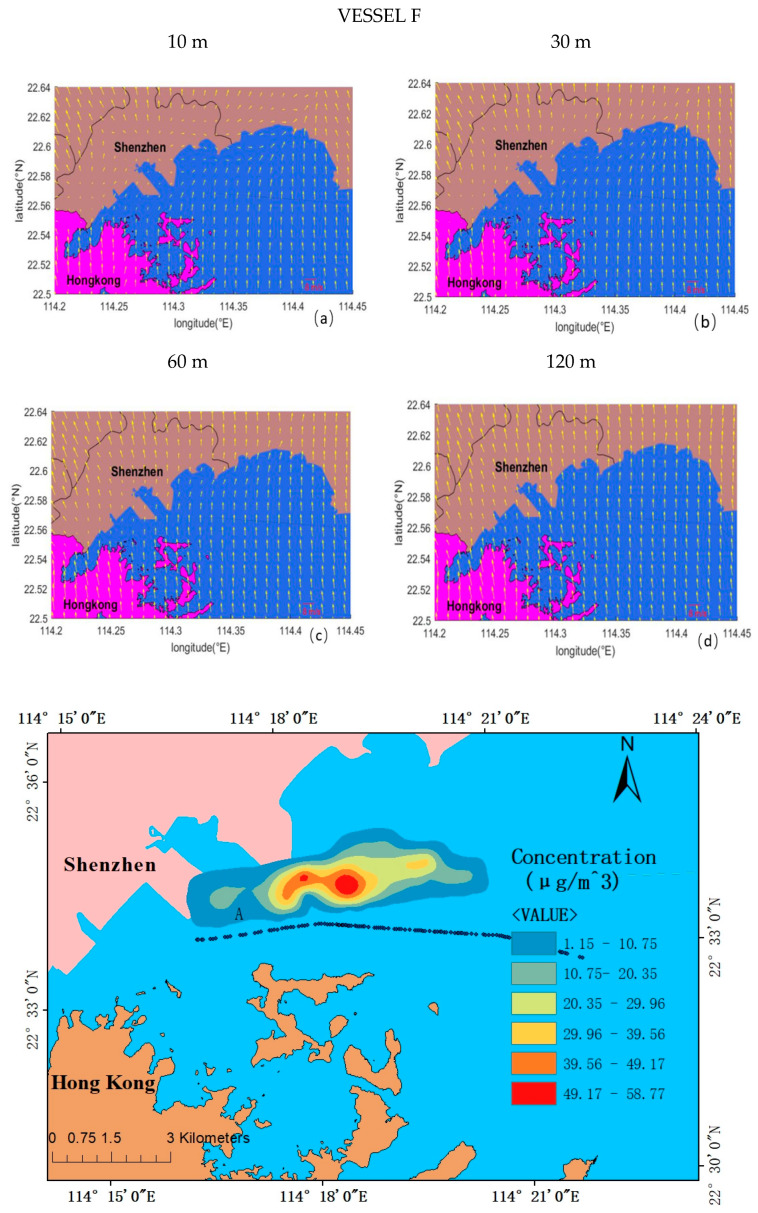
Wind field at different heights at the departure stage of Vessel F and the spatial distribution of the maximum concentration of each simulation grid during the simulation period. (**a**) 10 m, (**b**) 30 m, (**c**) 60 m and (**d**) 120 m.

**Figure A4:**
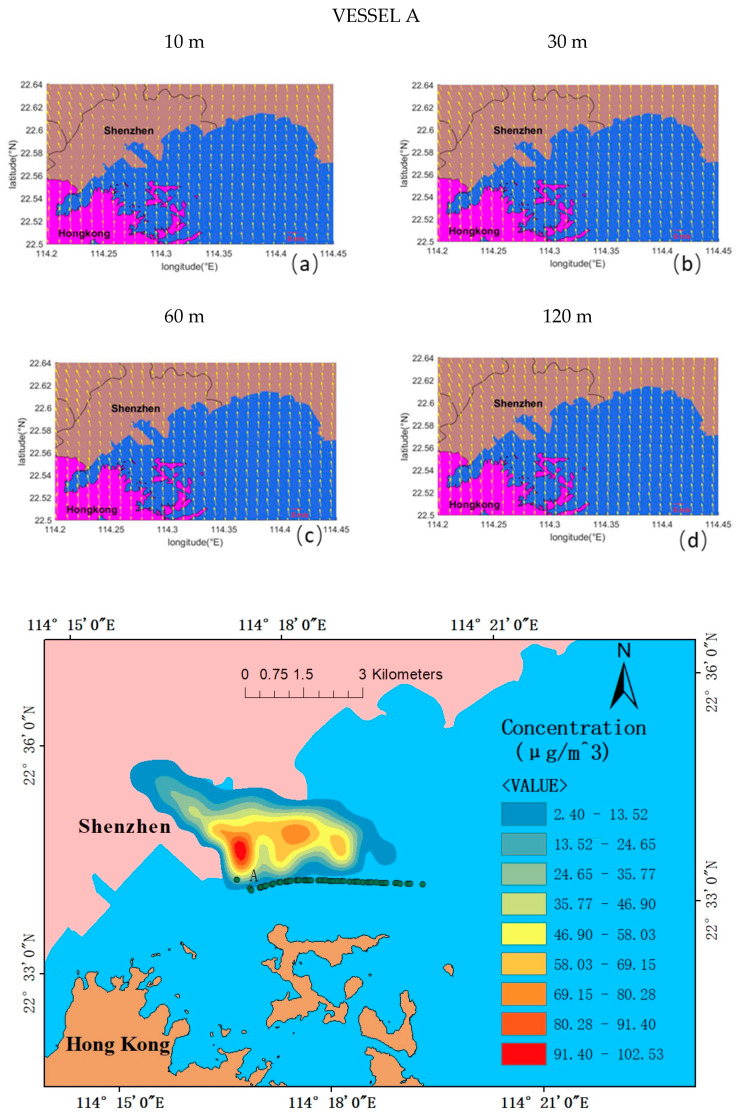
Wind field at different heights at the departure stage of Vessel A and the spatial distribution of the maximum concentration of each simulation grid during the simulation period. (**a**) 10 m, (**b**) 30 m, (**c**) 60 m and (**d**) 120 m.

**Figure A5:**
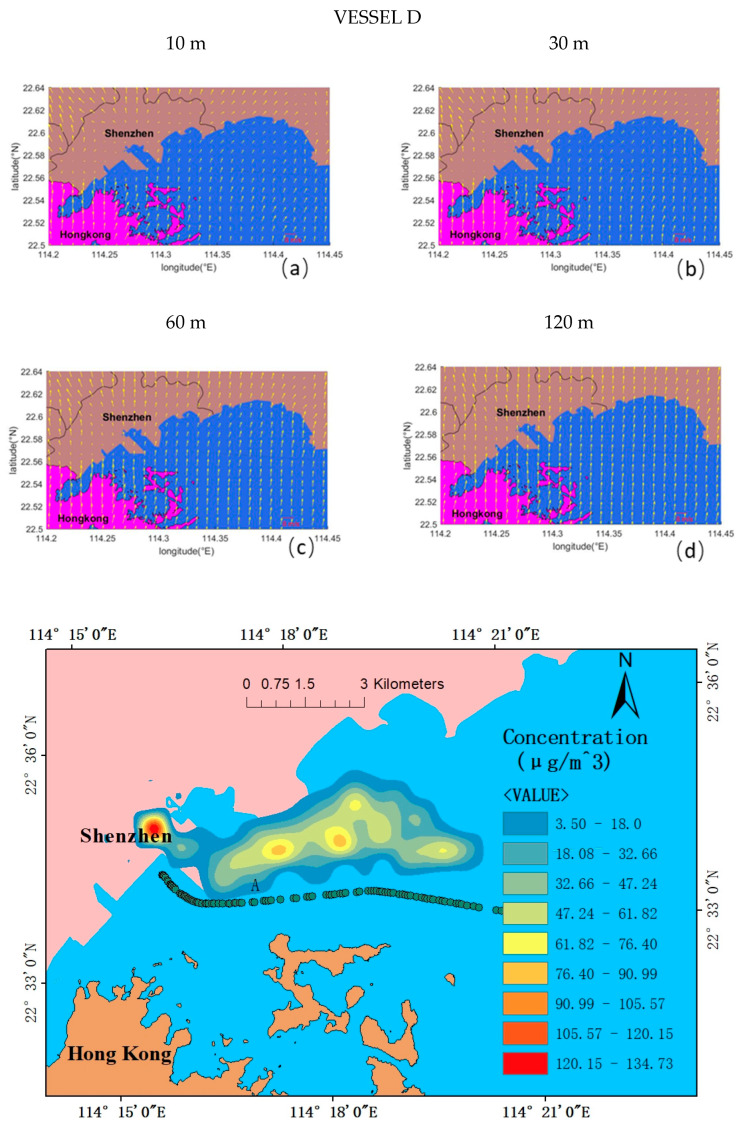
Wind field at different heights at the departure stage of Vessel D and the spatial distribution of the maximum concentration of each simulation grid during the simulation period. (**a**) 10 m, (**b**) 30 m, (**c**) 60 m and (**d**) 120 m.

**Figure A6:**
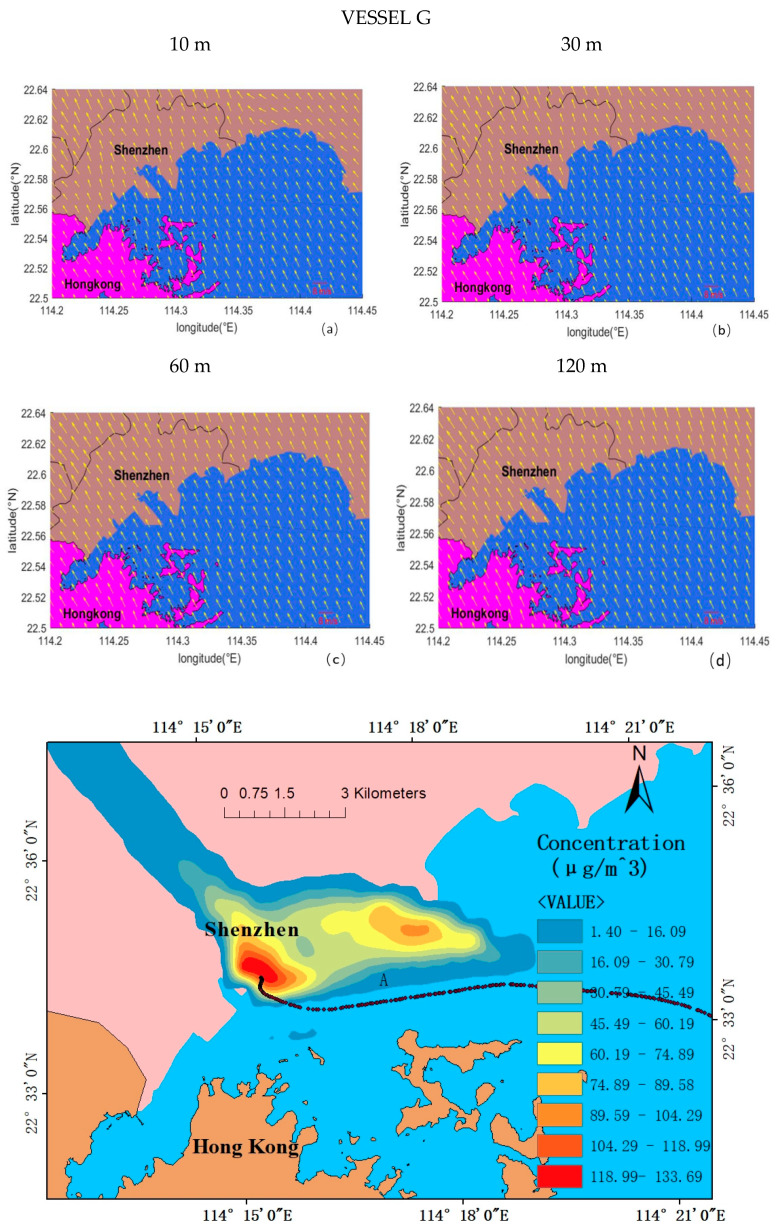
Wind field at different heights at the departure stage of Vessel G and the spatial distribution of the maximum concentration of each simulation grid during the simulation period. (**a**) 10 m, (**b**)3 0m, (**c**) 60 m and (**d**) 120 m.

**Figure A7:**
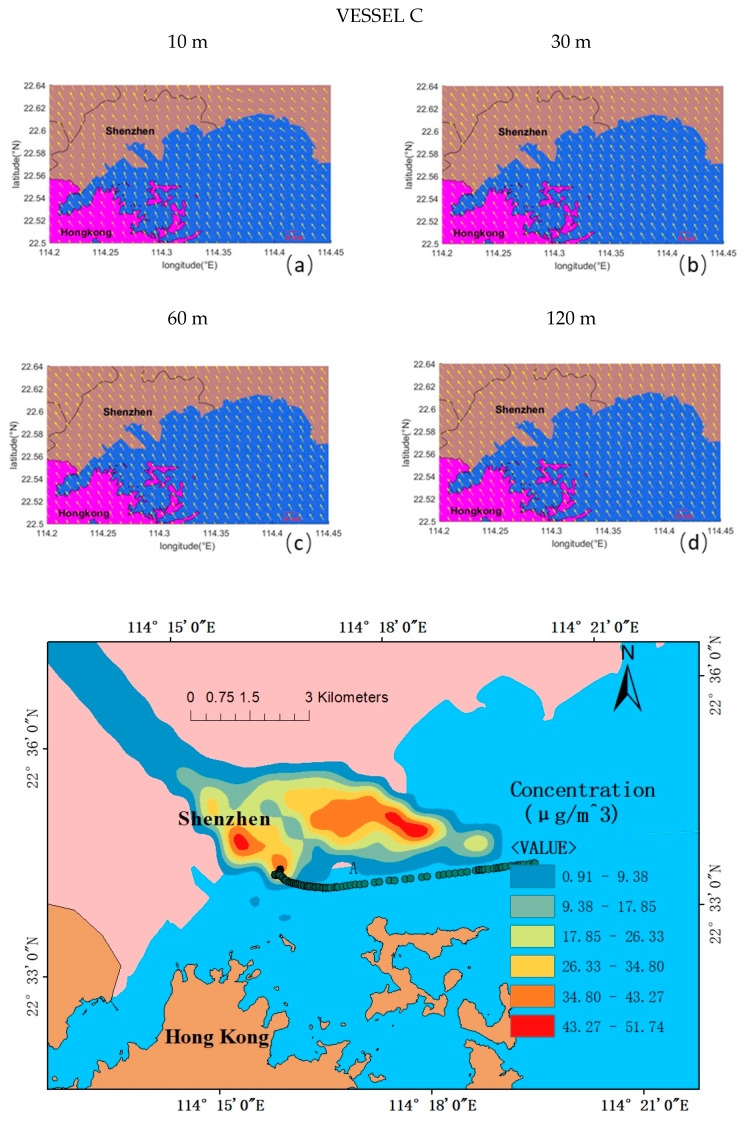
Wind field at different heights at the departure stage of Vessel C and the spatial distribution of the maximum concentration of each simulation grid during the simulation period. (**a**) 10 m, (**b**) 30 m, (**c**) 60 m and (**d**) 120 m.

**Figure A8:**
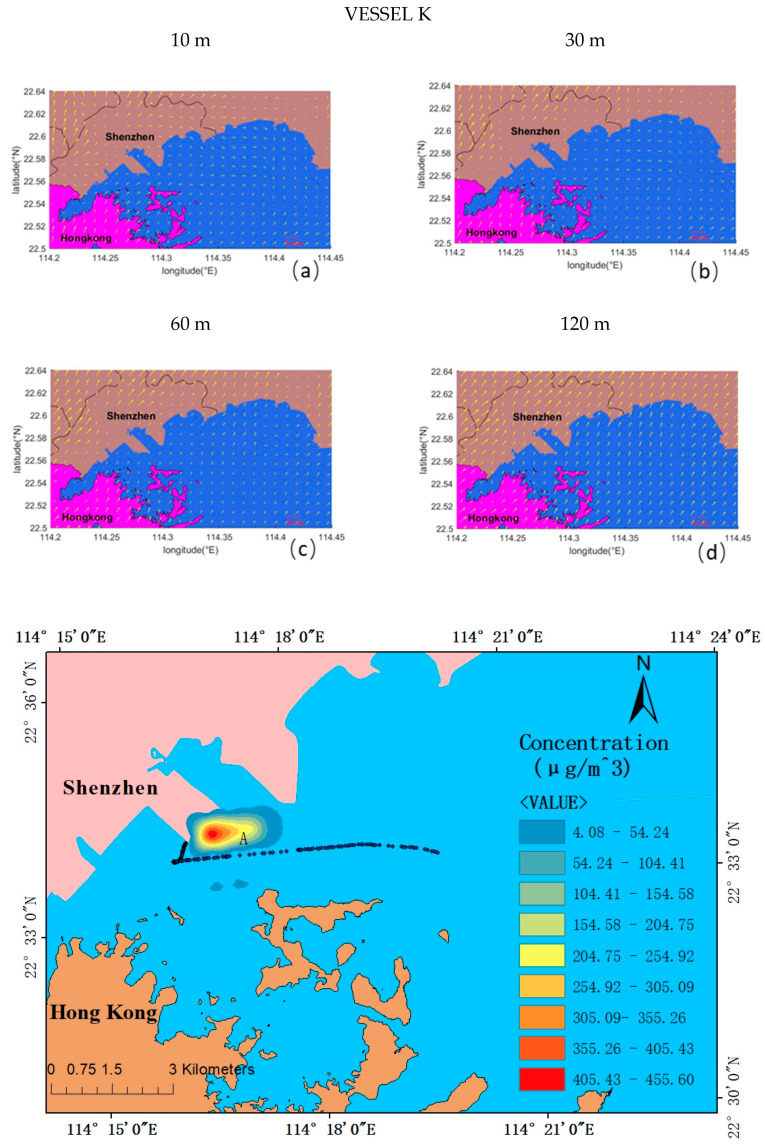
Wind field at different heights at the departure stage of Vessel K and the spatial distribution of the maximum concentration of each simulation grid during the simulation period. (**a**) 10 m, (**b**) 30 m, (**c**) 60 m and (**d**) 120 m.

**Figure A9:**
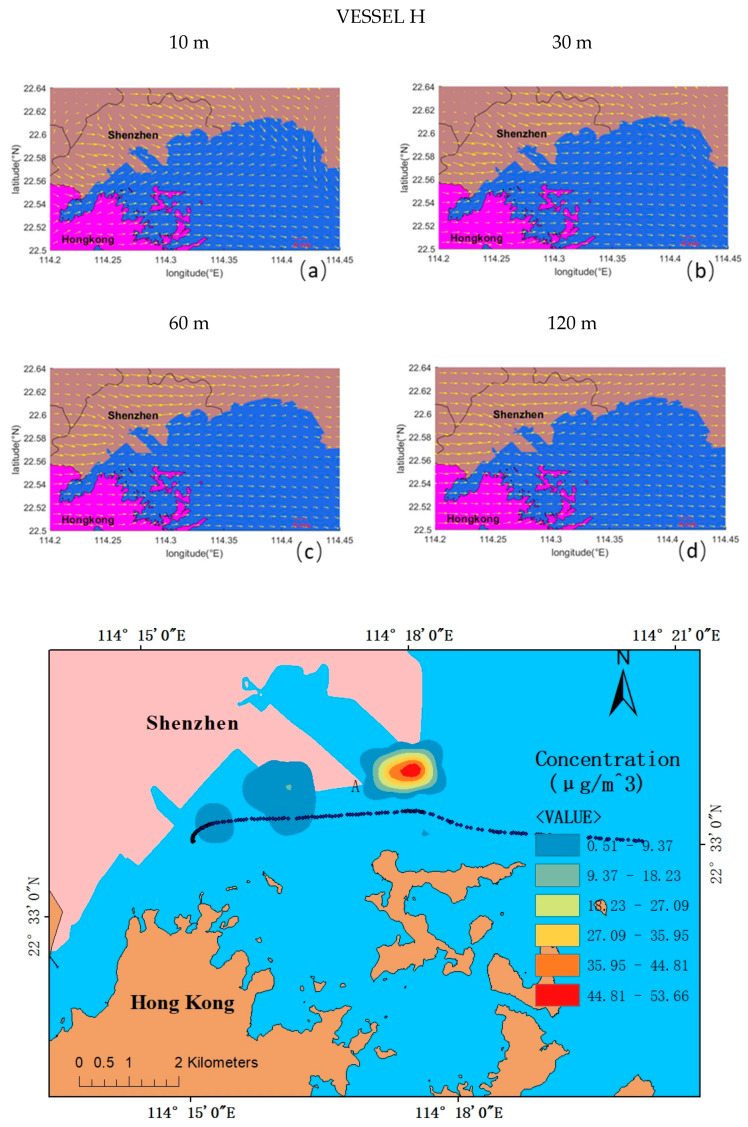
Wind field at different heights at the departure stage of Vessel H and the spatial distribution of the maximum concentration of each simulation grid during the simulation period. (**a**) 10 m, (**b**) 30 m, (**c**) 60 m and (**d**) 120 m.

**Figure A10:**
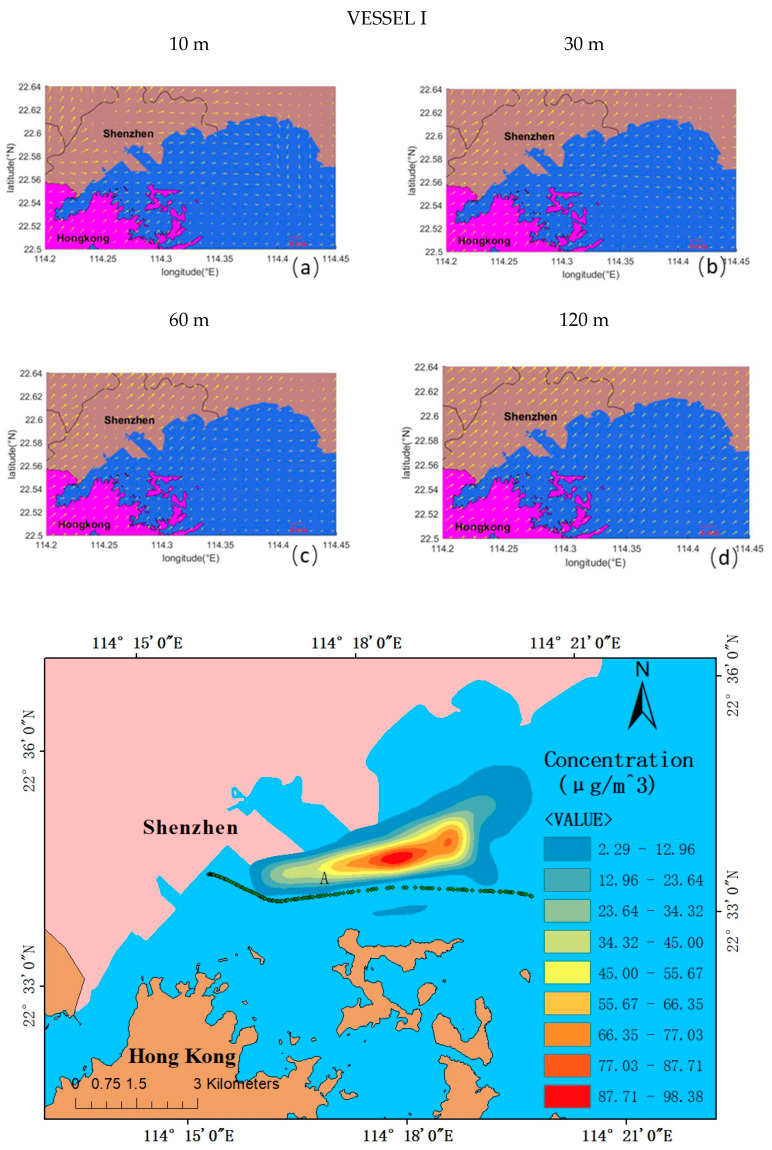
Wind field at different heights at the departure stage of Vessel I and the spatial distribution of the maximum concentration of each simulation grid during the simulation period. (**a**) 10 m, (**b**) 30 m, (**c**) 60 m and (**d**) 120 m.

**Figure A11:**
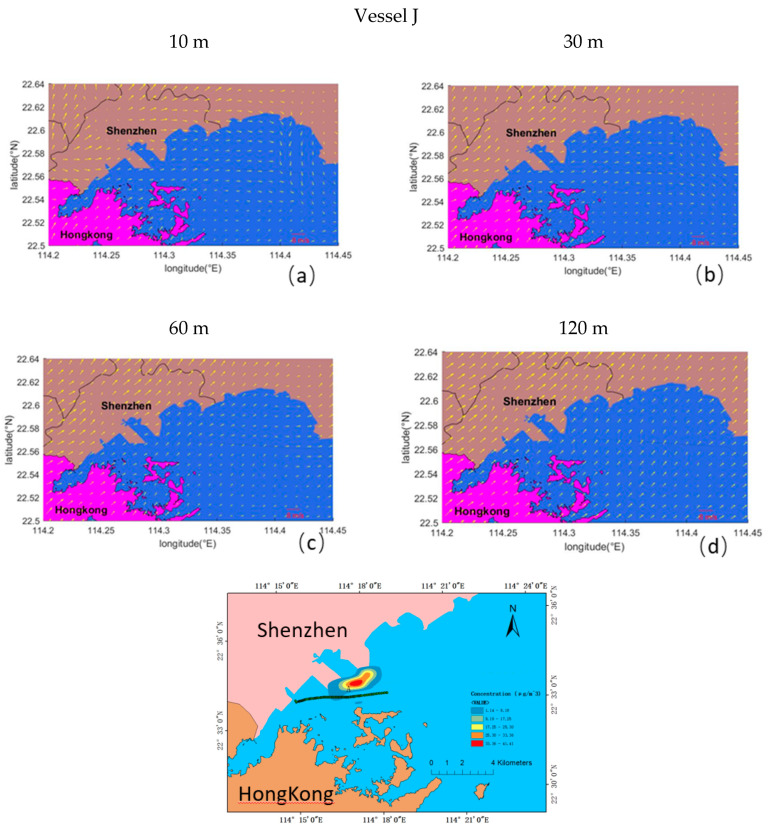
Wind field at different heights at the departure stage of Vessel J and the spatial distribution of the maximum concentration of each simulation grid during the simulation period. (**a**) 10 m, (**b**) 30 m, (**c**) 60 m and (**d**) 120 m.
